# Two Systems of Maintenance in Verbal Working Memory: Evidence from the Word Length Effect

**DOI:** 10.1371/journal.pone.0070026

**Published:** 2013-07-24

**Authors:** Gérôme Mora, Valérie Camos

**Affiliations:** 1 LEAD-CNRS, Université de Bourgogne, Dijon, France; 2 Département de Psychologie, Université de Fribourg, Fribourg, Switzerland; Harvard Medical School/Massachusetts General Hospital, United States of America

## Abstract

The extended time-based resource-sharing (TBRS) model suggested a working memory architecture in which an executive loop and a phonological loop could both support the maintenance of verbal information. The consequence of such a framework is that phonological effects known to impact the maintenance of verbal information, like the word length effect (WLE), should depend on the use of the phonological loop, but should disappear under the maintenance by the executive loop. In two previous studies, introducing concurrent articulation in complex span tasks barely affected WLE, contradicting the prediction from the TBRS model. The present study re-evaluated the WLE in a complex span task while controlling for time parameters and the amount of concurrent articulation. Specifically, we used a computer-paced span task in which participants remembered lists of either short or long words while concurrently either articulating or making a location judgment. Whereas the WLE appeared when participants remained silent, concurrent articulation eliminated the effect. Introducing a concurrent attention demand reduced recall, but did not affect WLE, and did not interact with concurrent articulation. These results support the existence of two systems of maintenance for verbal information.

## Introduction

Working memory (WM) is a system dedicated to the maintenance of information in the context of concurrent processing. In the seminal model of working memory by Baddeley [1], systems of maintenance were specific to either visuo-spatial or verbal domains (cf. also [2]). Shifting away from this structural view, other prominent theories, like that of Engle, Kane and Tuholski [3], suggested that maintenance is achieved through different strategies varying across tasks and individuals. In the first version of our model of WM, the time-based resource-sharing (TBRS) model, we favored the conception of a domain-general mechanism of maintenance, which depends on attention [4]. Recently, we proposed an extension to our model [5], [6], [7], in which a phonological loop can maintain a limited amount of verbal information through verbal rehearsal, while a central system, named executive loop, that can also maintain information by attentional refreshing. The aim of the present study was to bring further evidence on the existence of these two distinct systems of maintenance through the exploration of the word length effect (WLE) in complex span tasks.

In short-term memory literature, the WLE is defined by greater recall performance for lists of short words (e.g., *sum*, *hate*, *harm*, *wit*) compared to lists of long words (e.g., *association*, *opportunity, representative*, *organization*). Like the phonological similarity effect (PSE), the WLE has been considered as evidence for a domain-specific system of short-term maintenance for verbal information [1]. The WLE was attributed to subvocal rehearsal, which offsets decay of memory traces over time [8]. It was recently argued that WLE is not evidence for a time-based decay [9]. The current paper does not discuss the source of forgetting, but focuses on the fact that, whatever the source of forgetting, memory traces could be maintained through either a specialized phonological system or a central system. Because long words take more time to be pronounced, they are rehearsed at a slower rate than short words, and thus suffer from decay over longer periods of time. Consequently, these memory traces are weaker and more difficult to retrieve. Accordingly, Baddeley et al. [8] found that the duration of pronouncing words was the key factor that predicted the WLE. A second argument to supporting the rehearsal account is that the utterance of an irrelevant item (e.g., “the”) during the presentation of memoranda eliminates the WLE (e.g., [8], [10], [11], [12], [13], [14]). Such a repetition is assumed to prevent subvocal rehearsal on which the WLE relies.

Following the original explanation of the WLE put forward by Baddeley, numerous alternative accounts and corresponding evidence contradicting the original rehearsal account have been proposed. Cowan and collaborators [15] proposed that the differences in the duration of recall, or output delay, explained the WLE. Another explanation relied on the complexity of the items, sometimes combined with a discrimination account of memory [16], [17]. Alternatively, Hendry and Tehan [12] applied their idea of distinct processing of item and order information to account for the WLE. Recently, Jalbert, Neath, Bireta, and Surprenant [18] proposed that the WLE depends on differences in linguistic and lexical properties of short and long words rather than on length per se, such that a large neighborhood size disproportionately benefits short words. Finally, Campoy [19] showed that the WLE could be to some extent a consequence of retroactive interference. Although this is not an exhaustive list of all the accounts proposed for the WLE, they have all received support and criticism. Despite decades of study of the WLE in simple span tasks, a fully satisfactory understanding of the mechanisms underlying this effect is still lacking.

However, the aim of this paper is not to bring a further explanation or to provide support for or an argument against any of these accounts. Instead, this work focused on the idea that the two aforementioned systems of maintenance used for verbal information in working memory could affect the emergence of the WLE. This hypothesis has been never tested before. It should be noted that the nature of the most used paradigm *per se*, the simple span paradigm, could explain the rather complex state of affairs with a multiple of alternative accounts of the WLE. Indeed, the simple span paradigm requires the encoding of new memory items while maintaining the previously presented items in the list. As a consequence, encoding could be reduced by the concurrent maintenance, or the maintenance could be impeded because attention should be partially displaced to encode new memory items. The simple span paradigm does not allow a clear delineation between these possibilities. Moreover, in many studies, words have to be read aloud for further recall. This introduces a concurrent articulation that could affect both encoding and maintenance, and thus the simple span paradigm makes it impossible to distinguish the locus of the concurrent articulation effect. In contrast, the complex span paradigm has the advantage that encoding and maintenance are two distinct phases, thereby allowing experimental manipulations to specifically target one phase and not the other.

It should also be noted that, despite the diversity of the alternative accounts, the WLE remains a marker of the verbal nature of the memory traces. As a consequence, for models in which a domain-specific system of maintenance for verbal representations is embedded within a larger system [5], [20], the emergence of the WLE would depend on the reliance on this domain-specific system. We recently proposed such architecture for WM in our extended TBRS model [5], [6], [7]. The latest version of the multi-component model presents some similarities with our model [20]. In the TBRS model, WM is composed of a central system, named executive loop, in charge of both maintenance and transformation of WM representations. These representations are transient and built from information provided by sensory peripheral buffers and declarative long-term memory. Their maintenance is achieved by a general maintenance mechanism of attentional refreshing, which can reactivate decaying memory traces through attentional focusing. As a consequence, the maintenance of WM representations depends on the attentional demand introduced by a concurrent processing task. We have collected a large amount of evidence showing that recall performance decreases as the concurrent processing embedded in complex span task becomes increasingly demanding (for a review, [21]). We replicated this finding in the maintenance of verbal and visuo-spatial information [22]. We have also shown that the nature of the concurrent task (either verbal of visuo-spatial) does not affect the functioning of the executive loop, thus showing that this central system is not domain-specific [23], [24].

In addition to the executive loop, the TBRS model introduced a second loop, the phonological loop, which can maintain a limited amount of information through verbal rehearsal without the support of the central system. This creates an asymmetry between the maintenance of verbal and visuo-spatial information, such that verbal information can be maintained both by the executive and the phonological loops, whereas the visuo-spatial information is only maintained by the executive loop [23]. Such a framework also posits that the maintenance by the executive loop relies on multimodal representations through integrating input from the other buffers and declarative memory to build representations, whereas maintenance within the phonological loop depends on phonological codes only. The direct consequence of such framework is that the manipulations of phonological characteristics known for affecting maintenance of verbal information should depend on the use of the phonological loop, and should disappear under exclusive maintenance by the executive loop.

Previous studies have shown that the two maintenance mechanisms, refreshing and rehearsal, specific to the executive and phonological loops respectively, can operate independently from each other [7], [25]. Because of this independence, young adults can adaptively choose to use one or the other according to the constraints of the tasks or instructions [26]. These findings support the hypothesis of a functional autonomy of the two systems. However, they did not directly address the question that phonological effects would depend on the use of the phonological loop.

Recently, we tested this prediction through the PSE [27]. The PSE is characterized by better recall for phonologically dissimilar word lists compared with lists of phonologically similar words. Like WLE, it was frequently reported in simple span tasks and is considered as evidence of the phonological nature of memory traces. In a series of three experiments, Camos et al. [27] manipulated the opportunity to use either refreshing or rehearsal by varying the attentional demand of the concurrent task and inducing a concurrent articulation, respectively. Indeed, rehearsal does not depend on attention, at least, after a brief initial set-up period, [28], but on subvocal speech production that cannot operate concurrently with overt speech (i.e., concurrent articulation), whereas refreshing can operate concurrently with unrelated overt speech, but requires attention. Our study confirmed the previous work that observed the PSE in complex span tasks [26], [29]. Moreover, although both the attentional demand and the articulatory requirement of the concurrent task affected recall performance, only the induction of a concurrent articulation during maintenance eliminated the PSE. These results suggest that the emergence of the PSE in complex span tasks depends on the system used to maintain information. More specifically, the appearance of the PSE would require the maintenance of a phonological representation of memory items through rehearsal. This finding, added to the fact that the attentional demand of the concurrent task did not interact with concurrent articulation, favors models in which a domain-specific system of maintenance is embedded among a larger system [5], [20]. The aim of the present study was to extend this finding by testing another well-known phonologically-related effect, the WLE, using complex span tasks that similarly varied the opportunity for rehearsal and refreshing.

Contrary to the large amount of studies in simple span tasks, the WLE has been rarely studied in complex span tasks. This is all the more surprising because performance in complex span tasks is a better predictor of high level cognition than performance in simple span tasks (e.g., [30], [31]). As a consequence, it is a widely used tool in psychology that should be systematically explored to define what could affect human behavior in this task. Concerning the WLE and its potential impact on recall, only two studies explored this effect in complex span tasks [32], [33], leading to rather unexpected findings.

La Pointe and Engle [32] compared recall of one-syllable vs. three to four-syllable words in complex span tasks (i.e., reading and operation span tasks) with a simple span task. They found the short word advantage on free recall for all the tasks of the first two experiments. The nature of the concurrent tasks (either reading sentences or verifying operations) in complex span tasks did not affect the size of the WLE, but the effect was much smaller compared to the simple span task. It should be noted that, both in reading and operation span tasks, participants had to read sentences and operations aloud, adding then a concurrent articulation to the attentional demand of the task. In the Experiment 3, the authors introduced a concurrent articulation by asking participants to repeat continuously “abcabc” aloud. The concurrent articulation did not make the WLE disappear in either operation or simple span tasks, which contradicts the previous studies (e.g., [8], [10], [11], [12], [13], [14]). The size of the WLE was reduced in both span tasks compared to Experiment 2 and reached similar values for simple and complex span tasks. However, the level of recall was rather low for both tasks, around 15% for long word lists, and the reduction of the standard deviation values may indicate a floor effect. Such findings were replicated in their Experiment 4, which compared operation span tasks with and without concurrent articulation. The disappearance of the WLE by concurrent articulation occurred only when a closed set of words replaced the open set used as memoranda in the previous experiments. The authors suggested that a deeper or different kind of code is used for the words when the set is unlimited.

Apart from the fact that such code remains to be defined, another weakness of this study is that these findings were not replicated by Tehan, et al. [33]. In their study, Tehan et al. [33] compared the WLE in a simple span task and two complex span tasks using a fixed set of words. These complex span tasks were an operation span task, which required subjects to read and verify operations silently, and a reading digits span task using the same material as the operation span task but the digits were read aloud, thereby inducing a concurrent articulation. Replicating La Pointe and Engle [32], Tehan et al. [33] showed the WLE in the operation span task as in the simple span task. However, and contrary to Experiment 4 from La Pointe and Engle [32], the WLE also occurred in the reading digits span task with a similar size as in the operation span task. Overall, the induction of a concurrent articulation did not eradicate the WLE in complex span task. This finding led Tehan et al. [33] to conclude that the WLE should no longer be considered as signature of the operation of a phonological loop, but of performance over short-term retention.

To summarize, two studies showed the WLE complex span tasks, but their findings were divergent. Moreover, neither study’s design disentangled the concurrent articulation and attentional demand of the secondary task to better understand respective impact of the two maintenance mechanisms available for verbal information. For example, in Tehan et al. [33], the concurrent task was either a highly demanding but silent task or a low demanding task performed aloud. Another important characteristic of the tasks used in the two reported studies is that all tasks were self-paced. Although the experimenter pressed a key to make items appear on screen and instructed participants to perform tasks as soon as information appeared on screen, participants performed the concurrent tasks at their own pace. Without control of time parameters, it is impossible to ensure that participants did not save some milliseconds to refresh memory items. For example, it was shown that it takes about 40 ms to refresh one memory item, something that remains totally unnoticeable without strict time control [34]. Moreover, Camos et al. ([7], see also [4]) showed that the pace of concurrent articulation affects recall performance in complex span tasks. Difference in the pace of concurrent articulation could account for the difference in results between Experiments 2 and 3 from La Pointe and Engle [32], as the continuous repetition of “abcabc” may induce more articulations than reading operations. Due to lack of information about the different paces of articulation, it is difficult to verify this supposition. Nevertheless, this emphasizes how important it is to control the number of articulations produced per time unit and more generally the duration of the concurrent task. To conclude, the two sole studies testing the WLE in complex span tasks did not replicate each other or previously observed effect of concurrent articulation. We suggest that the poor control of factors known for affecting maintenance at short term may explain the incongruent results.

The aim of the present study was thus to explicitly test the impact of articulatory suppression and attentional demand induced by the secondary task of complex span tasks on the WLE while time parameters were controlled. Specifically, we used the computer-paced span tasks we designed to test the TBRS model, in which the duration of the processing episodes in the complex span task are the same across conditions. The attentional demand of the secondary task and the presence of a concurrent articulation during maintenance were orthogonally manipulated to impede attentional refreshing and subvocal rehearsal, respectively. The concurrent attentional demand was a silent location judgment task in which participants were to judge the location of a square presented either on the bottom or top of the screen by pressing designated keys. This visuo-spatial task with manual responses was intended to minimize sources of potential representation-based interference. Although the aforementioned studies regarding the WLE in complex span tasks used verbal concurrent tasks (e.g., reading digits or sentences; [32], [33]), verbal distractors could interfere with verbal memoranda (i.e., words) and obscure the effects of the two factors of interest here. The concurrent articulation was induced by asking participants to repeat “oui” (yes). The articulation of this very frequent and monosyllabic word impedes the phonological mechanism during maintenance, but it does not require much attention [28]. To control for the amount of articulation and to keep its pace constant across conditions and participants, participants heard a series of beeps in a headphone and said “oui” for each beep. Although it might be suggested that hearing tones would have a detrimental effect on recall, Jones and Macken [35] have shown that the repetitive presentation of the same tone during maintenance does not affect recall performance compared with a quiet condition. Finally, to avoid potential proactive interference that may obscure the effect of our experimental manipulations, we chose to use an open set of words to create the lists of 6 short and long words that participants memorized.

As predicted and observed with PSE [26], we expected that the WLE in a complex span task would depend on the use of the phonological loop. As a consequence, whereas the attentional demand of the concurrent task and the induction of a concurrent articulation should both reduce recall performance, only the concurrent articulation should eliminate the WLE.

## Methods

### Ethic Statement

The study was performed in a pedagogical context, in which students can participate in a non-invasive laboratory experiment in exchange for course credits. The study was anonymous, conducted in accordance with the ethical standards set out in the 1964 Declaration of Helsinki, and approved by the institutional review board of the Laboratoire d'Etude de l'Apprentissage et du Développement. All participants gave written informed consent.

#### Participants

Thirty-six undergraduate students at the University of Bourgogne received partial course credit for participating. The 26 women and 10 men were all French native speakers, aged between 17 and 24 (*M* = 19.90, *SD* = 1.37). None of them participated in the pre-test described in the supporting information.

### Materials and Procedure

Because the WLE has never been investigated in French, we built lists of short and long words. Two sets of 96 singular French nouns were each selected from Lexique 3 database [36]. One set was made of short words that were monosyllabic words with three phonemes and four letters. The other set was made of long words that were disyllabic words with five phonemes and six letters. Both sets had close frequencies, degree of concreteness and imageability, and they induced the WLE in a simple span task (cf. pretest S1).

**Figure 1:**
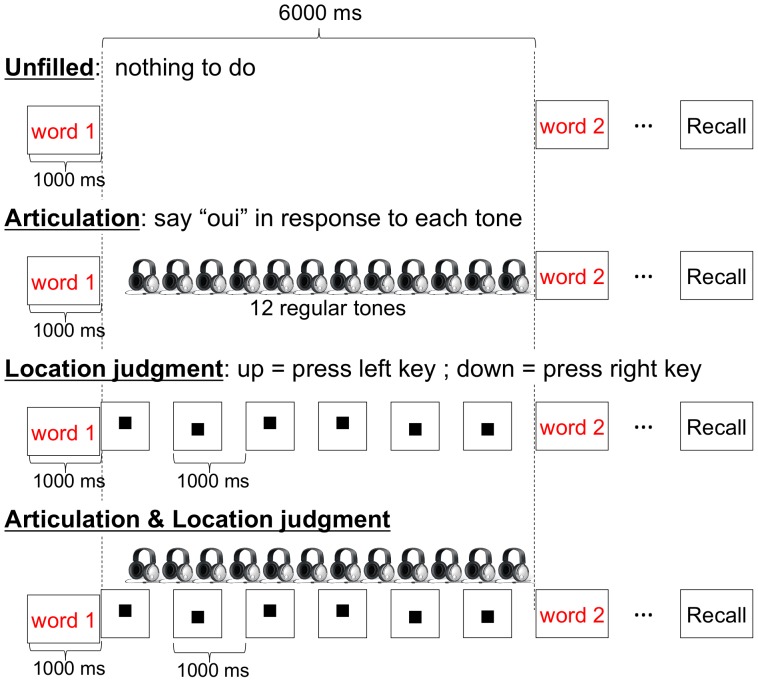
Illustration of the four conditions.

As in the pre-test, a series of six words were drawn at random without replacement from each set. Participants were seated at about 60 cm from a computer screen, which displayed the tasks using Psyscope software [37]. Participants were presented with four blocks arranged in randomized order. For each block a complex span paradigm was used, but the concurrent task varied between blocks. Each word to remember was followed by a 6000 ms delay. Depending on the block, the after-word delay was either filled with an articulation task, with a location judgment task, with both articulation and location judgment tasks, or remained unfilled (Figure 1). For the articulation task, a series of twelve 10 ms tones (32 bits, 44100 Hz) interleaved with 490 ms silence periods was displayed through a headphone. The first tone appeared 500 ms after a word to remember disappeared. Participants were instructed to say “oui” each time they heard the tone. For the location judgment task, a series of six black squares (2 cm side) was displayed on the computer screen. Each square appeared 666 ms and a blank screen was interleaved between squares for 334 ms. The first square appeared immediately after the word to remember disappeared. Each square randomly appeared in either the lower or the upper part of the screen (1.5 cm apart from the middle of the screen) with the same frequency. Participants were instructed to press a key on the right side or on the left side of a keyboard each time the square appeared in the lower or the upper location respectively. In the block involving both articulation and location judgment tasks, participants were simultaneously presented with the two tasks described above. For the unfilled delay block, nothing was displayed neither through headphone nor on screen during the 6000 ms delays.

Each block started with practice trials followed by eight testing trials. For the unfilled, and concurrent articulation, and location judgment blocks, participants received one practice trial to familiarize themselves with this task, and received two more trials in the block involving both simultaneous articulation and location judgment tasks. In practice trials, words were replaced by forenames to avoid any interference with the testing words. Within each block, eight testing trials were presented, half with long words and half with short words. The order of the eight trials was randomized in such a way that trials of a certain length of words could not be displayed more than twice in a row. Words within a trial were drawn at random without replacement from short or long word sets, and thus each word was displayed only once during the overall experiment. Each trial began with an asterisk centered in the screen for 500 ms followed by a first word presented in red for 1000 ms. After the 6000 ms filled or unfilled delay, the second word appeared for 1000 ms, and so on. Immediately after the end of the last after-word delay, recall signal (i.e., “Rappel”) appeared on screen prompting participants to recall the words in the same order as words were presented. Then, 1000 ms afterward, “1:” appeared on the screen, indicating participants to type the first word on keyboard. After they finished typing the first word, they pressed “Enter” to go to the second word, then “2:” appeared on the screen, and so on. If participants were not able to remember a word, they just pressed “Enter” to go to the next one. They were informed that they could not go back to previous words after “Enter” had been pressed. Then, participants pressed the space bar to start the next trial. Response times and accuracy were recorded for the location judgment task, and for the articulation task, the experimenter counted the number of “oui” uttered by participants during each after-words delay. The experiment lasted about one hour.

## Results

Three participants failed to reach 80% correct responses to the location judgment task and five others failed to articulate “oui” at least ten times on average during the articulation tasks. Nevertheless, discarding these eight participants did not impact the pattern of results, thus we report the results including the overall sample. To ensure that variation of memory performance could not be attributed to variation of performance in articulation task or location judgment task, we analyzed the number of utterances, and percentage of correct locations, as well as RTs. Performance did not significantly differ during memorization of long or short words in terms of location accuracy (87% for both long and short words), location RTs (respectively 417 ms and 412 ms) or number of utterances (11.2 for both long and short words), *p*s >.05. The articulation task did not influence location performance (88% and 408 ms without concurrent articulation versus 86% and 421 ms with concurrent articulation), *p*s >.05. However, participants tended to underperform the articulation task when concurrently performing the location judgment task (on average 10.4 “oui” uttered versus 11.9 without concurrent location task), *F*(1,30) = 30.07, *p*<.001, η^2^_p_ = .50.

**Figure 2:**
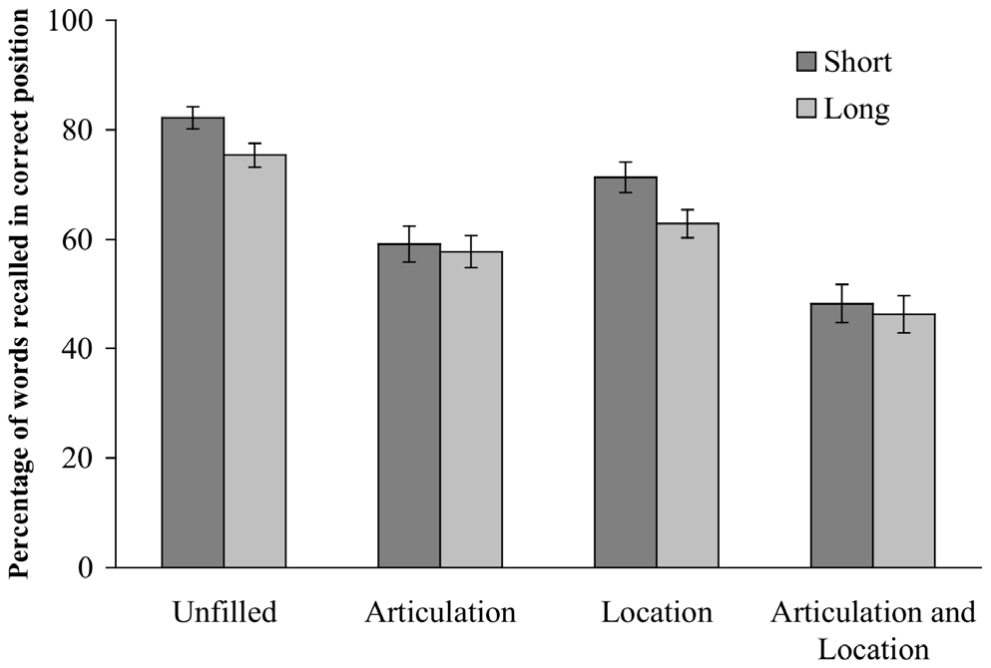
Percentage of words recalled in correct position for short and long words as a function of concurrent task to perform.

Recall performance was scored as the proportion of words recalled in correct position. An analysis of variance was performed with the length of words (long *vs*. short), the occurrence of the location judgment task (with *vs.* without) and the presence of a concurrent articulation (with *vs.* without) as within-subject factors. The analysis revealed three main effects. First, both the occurrence of the location judgment task and of the concurrent articulation reduced recall performance, from 69% to 57% for concurrent location judgment task, *F*(1,35) = 37.76, *p*<.001, η^2^_p_ = .52, and from 73% to 53% for concurrent articulation task, *F*(1,35) = 84.79, *p*<.001, η^2^_p_ = .71. Second, short words (65%) were better recalled than long words (61%), *F*(1,35) = 14.90, *p*<.001, η^2^_p_ = .30. The occurrence of the location judgment task did not interact with length of words to remember or with the presence of concurrent articulation, *F*s <1. However, the interaction between WLE and concurrent articulation was significant, *F*(1,35) = 5.98, *p*<.05, η^2^_p_ = .15 (Figure 2). Recall was greater for short than long words in conditions involving no articulation, namely, the unfilled condition (82% and 75%, respectively), *F*(1,35) = 6.50, *p*<.05, η^2^_p_ = .16, and the location judgment task condition (71% and 63%, respectively), F(1,35) = 8.98, *p*<.01, η^2^_p_ = .20. However, the WLE was not significant in conditions with concurrent articulation, *F*s <1 (respectively 59% and 58% for short and long words in articulation condition, and 48% and 46% in condition involving both articulation and location judgment tasks). The three-way interaction was not significant, *F* <1.

Because the score in correct-in-position does not distinguish the maintenance of item vs. order information, recall performance was scored in two different ways, considering the proportion of words recalled regardless position (*correct item*) and the proportion of words recalled in correct position (the performance measure used in the previous analysis) as a function of words recalled regardless position (i.e., correct-in-position score/correct item score), called *order accuracy*. As we did for correct-in-position score, an ANOVA was performed on each of these two scores. The overall pattern for these two scores replicated our results described previously using the correct-in-position score. The occurrence of the location judgment task (89% vs. 82%, *F*(1,35) = 18.01, *p*<.001, η^2^_p_ = .34, for order accuracy, and 76% vs. 68%, *F*(1,35) = 26.78, *p*<.001, η^2^_p_ = .43, for correct item) and of the concurrent articulation (90% to 81%, *F*(1,35) = 19.11, *p*<.001, η^2^_p_ = .35, and 80% vs. 63%, *F*(1,35) = 94.40, *p*<.001, η^2^_p_ = .73, respectively) reduced recall performance for both scores. Short words were better recalled than long words (88% vs. 84%, *F*(1,35) = 10.45, *p*<.01, η^2^_p_ = .23) for order accuracy, although this effect (73% vs. 71%, *F*(1,35) = 3.38, *p* = .07, η^2^_p_ = .09) just failed to reach significance when using the correct item score.

The occurrence of the location judgment task never interacted with the length of memory words or with the concurrent articulation, *F*s <1. The interaction between word length and concurrent articulation exhibited the same pattern as in the previous correct-in-position analysis. Specifically, the WLE was never significant in conditions involving a concurrent articulation, i.e., in the articulation condition (86% vs. 84% for order accuracy, and 67% vs. 68% for correct item score) and in the condition with both articulation and location judgment tasks (78% vs. 76%, and 60% vs. 59%), *F*s <1. However, short words were better recalled than long words when the task was silent, i.e., in the unfilled condition (95% vs. 91%, and 86% vs. 82%), *F*(1,35) = 4.65, *p*<.05, η^2^_p_ = .12, and *F*(1,35) = 4.12, *p*<.05, η^2^_p_ = .11, respectively, and in the location judgment condition (91% vs. 83%, and 78% vs. 75%), although this effect failed to reach significance for the correct item scoring, *F*(1,35) = 10.80, *p*<.01, η^2^_p_ = .24, and *F*(1,35) = 1.60, *p* = .215, η^2^_p_ = .04, respectively. The three-way interactions were not significant for all scoring methods, *F*s <1.

## Discussion

The aim of the present study was to test a proposal issued from our model of working memory, which is also congruent with the last version of the multi-component model [5], [7], [20]. Our model includes a specific system for the maintenance of verbal information under verbal code, the phonological loop, in addition to a more general attention-dependent system of maintenance, the executive loop. As a consequence, the impact of the phonological characteristics of memory items should depend on the use of the verbal-specific system. Conversely, recall performance should be immune from phonological effects under the use of the executive loop. To vary the implication of each system on the maintenance of word lists, we impeded the executive and phonological loops by adding either a secondary attentionally demanding task or a concurrent articulation, respectively. Four findings that were replicated in three scoring methods emerged from this study.

First, as predicted and confirming results from LaPointe and Engle [32] and Tehan et al. [33], we observed a WLE in a complex span paradigm, with short words being better recalled than long words. This effect, which is considered as a benchmark in short-term memory literature, was often observed in simple span tasks, but poorly explored in complex span tasks. It should be noted that the changes in the complex span paradigm between the two previous studies and the current one do not seem to affect the WLE. For example, we used a computer-paced task with visuo-spatial stimuli in the secondary task while previous studies used an experimenter-paced paradigm in which the distractors were verbal (i.e., sentences, operations or digits). Thus, the present study extends the occurrence of the WLE in complex span tasks involving a variety of procedures and distracting tasks.

The second main finding was that the WLE disappears when the verbal-specific system is impeded by a concurrent articulation. This result was replicated in two conditions and is in accordance with our hypothesis that the emergence of the WLE depends on the maintenance of verbal information in a verbal-specific system through subvocal rehearsal. LaPointe and Engle [32] also reported the disappearance of the WLE under concurrent articulation. However, this phenomenon was restricted to the use of fixed pool of words. With open sets, the WLE remained under concurrent articulation. Although this last finding may be at odds with the current study in which we used an open pool of words, scrutiny of LaPointe and Engle’s [32] results reveals that they observed a trend congruent with our proposal. Indeed, in three experiments, they used the same open pool of words in an operation span task, but they varied the use of concurrent articulation across experiments. When operations were read silently, the WLE was large and recall difference between short and long words was 15%. When participants had to read operations aloud, the WLE was reduced (11%), and even more reduced (5%) during the continuous utterance of “abcd”. Although this last difference remained significant, the introduction of a concurrent articulation (i.e., reading operations) reduced the WLE, which became much smaller under dense articulation (i.e., continuous utterance of “abcd”). The overall pattern of LaPointe and Engle’s [32] results showed that the more the verbal system is impeded by concurrent articulation, the less likely phonological characteristics affect recall, as we predicted and observed in the present study. Moreover, it is not possible to conceive that the reduction of the WLE relied on increased representation-based interference produced by the articulation. Reading operations lead to utter between 7 and 14 different syllables [cf. the examples given by LaPointe and Engle, 32], which should produce a substantial level of interference with the memory items compared with the 4 syllables “abcd”.

Whereas LaPointe and Engle [32] reported findings that are congruent with the present results, it remains that Tehan et al. [33] did not observe either an elimination or even a reduction of the WLE under concurrent articulation. Contrary to what we did for LaPointe and Engle [32], it is not possible to compare across experiments for Tehan et al. [33], because different secondary tasks were employed. The only difference that may have some influence on the discrepancy between the results is the lists of words. For sake of comparison, Tehan et al. [33] used the same lists of 8 short and 8 long words as Baddeley et al. [8]. Is the effect observed by Tehan et al. [33] restricted to this set of words? As noted in several publications, it has become increasingly apparent that the particular word set used can critically determine whether an effect occurs (e.g., [9], [18], [38]). To avoid such an issue, the present study, as LaPointe and Engle [32], used open sets.

The elimination or reduction of the WLE under concurrent articulation does not imply that verbal items could only be maintained by a domain-specific system. The reduction of available attention by a concurrent location judgment task reduced recall of verbal items, suggesting that a second system relying on attention-demanding processes could participate to the maintenance of verbal information. However, and this is our third finding, WLE was left unchanged by this manipulation of attention. This attention-based system would therefore maintain verbal information under a format that minimizes the impact of the phonological characteristics. Although our study did not indicate the exact nature of the memory traces maintained under this system, some speculations are possible. Lesion studies as well as functional neuroimaging bring convergent evidence in favor of two distinct neural networks underlying verbal short-term memory (for a review, [39]). For example, Hanten and Martin [40] distinguished one network involving the superior temporal lobe and the supramarginal gyrus that would subserve in the retention of phonological information from another network constituted by the inferior and middle temporal lobe and the inferior frontal lobe that would maintain semantic information. Accordingly, patients with damage to left inferior and middle frontal gyri show deficits in semantic short-term memory (STM), while damage to inferior parietal areas is associated with deficits in phonological STM [41]. While further studies are needed to exactly delineate these two networks, phonological and semantic STM appear to be related to different patterns of brain damage and neural activation. It might be suggested that the verbal-specific system we hypothesize in our model would map on the phonological network described above, because we showed that the WLE as well as the PSE appear when this system is available and disappear when it is not. On the other hand, further studies are needed to characterize the nature of representations manipulated by the attention-based system.

Finally, the addition of a secondary task on the one hand and of a concurrent articulation on the other hand had no interactive effect on the WLE, although each of these factors reduced recall performance as expected. This replicates previous findings and is congruent with the proposal of two distinct systems, as suggested in Camos et al. [7] or Baddeley [20].

The present findings support our predictions that the WLE in complex span tasks depends on the availability of a verbal-specific system of maintenance, which could be impeded by concurrent articulation. However, it was recently suggested that concurrent articulation does not reduce recall through the impediment of subvocal rehearsal, but rather it introduces supplementary verbal material that could interfere with the memory items. To impede subvocal rehearsal, the traditional technique requires asking participants to overtly articulate words differing from the memory items. It could then be suggested that the pronounced words could induce representation-based interference, which depends on the novelty of these pronounced words and the degree of overlap (or similarity) between them and the memory words. In the SOB model, forgetting results from interference produced by the encoding of additional information [42]. The encoding strength of an item is a function of its novelty with the current content of short-term memory, with novel items being encoded with a large encoding weight. As a consequence, when distractors are interspersed between retrievals in serial recall tasks, repeating the same distractor several times does not produce further forgetting, whereas additional forgetting is observed when the nature of the distractors to be uttered is changed [43], [44]. The present study presented a situation similar to the former, as participants had to repeat the same word “oui” all along the trials. Moreover, the role of novelty in forgetting was strongly challenged in a recent series of experiments that failed to observe any effect of the novelty of distractors on recall [45]. Concerning the degree of similarity between “oui” and the memory items, the first phoneme [w] in “oui” did not occur in any other words of the lists. The second phoneme was shared by more words in the long word pool than in short word pool (28 vs. 12), which is an obvious consequence of the fact that more phonemes are represented in long than in short words, the phoneme [i] being rather frequent in French. As a consequence, if the pronunciation of “oui” induced representation-based interference, this interference should be stronger in long words, reinforcing the WLE. On the contrary, we observed in the present study that concurrent articulation eliminated the WLE.

Moreover, previous studies have shown that the overlap of phonemes between memory items and distractors leads to a rather small detrimental effect on recall performance. When contrasting a low overlap condition in which no phoneme was shared between memoranda and distractors to a fully overlap condition in which all the phonemes of memoranda were shared by distractors, Oberauer [46] reported a reduction of 6% of recall. In the present study, although only 4% and 6% of the phonemes of the short and long words, respectively, were shared with “oui”, this concurrent articulation resulted in a reduction of 20%. Thus, the type of concurrent articulation we used in the present study most probably reduced recall performance because it impeded some mechanism instead of creating interference with the memory items.

To conclude, the present study brought further evidence of the distinction between two systems of maintenance of verbal information in working memory, a domain-specific system and a general attention-based system (see also [7], [26], [27]). It replicated with the WLE the pattern of findings observed by Camos et al. [27] with the PSE. Thus, the phonological characteristics of memory items affect recall only when the domain-specific system of maintenance can be used, on which the emergence of the WLE and PSE is dependent.

## Supporting Information

Pretest S1 **Method and results of a pre-test, which allowed creating the lists of memory items.**
(DOC)
