# Supplementary material for: Two Systems of Maintenance in Verbal Working Memory: Evidence from the Word Length Effect
Source: PLoS One. 2013 Jul 24;8(7):e70026. doi: 10.1371/journal.pone.0070026 (PMC3722204; doi:10.1371/journal.pone.0070026)
Supplement: Pretest S1 — Method and results of a pre-test, which allowed creating the lists of memory items. (DOC) [file pone.0070026.s001.doc]

**Supporting Information**

According to Lexique 3 database, short and long word sets had close frequencies: 58 (ranging from 8 to 369) and 51 (from 10 to 355) million occurrences in books for short and long words sets, respectively, *t*(95) = 0.63, *p* = .49. We evaluated concreteness and imageability of the words on 43 French native-speakers (8 males; *M* = 20.80; *SD* = 1.83), who did not participate in either the experiment or the pre-test. The procedure was similar to Bonin et al. (2003) to allow comparisons. Participants received 2 lists of words, in which the short and long words were randomly presented in a different order for each list. On a 5-point Likert scale, they had to score the concreteness of the words for one list and the imageability on the other list. The order of presentation of the lists was counterbalanced across participants. Short and word longs did not differ on concreteness (3.58 vs. 3.60), and imageability (3.59 vs. 3.63), *t*(190)=.16, *p* = .88 and *t*(190) = .23, *p* = .82, respectively. Some of the words (51%) were previously tested for concreteness and imageability by Bonin et al. (2003). On this subsample of words, our evaluation strongly correlated with the values observed by Bonin et al. (2003), *r*s = .66 and .69, respectively.

We tested these sets of words using a simple span task to ensure it produced the classic WLE. Fourteen undergraduate students received partial course credit for participating in this pre-test. The 14 women were all French native speakers, aged between 17 and 23 (*M* = 19.10; *SD* = 1.49). A series of six words was drawn at random without replacement from each set and was successively displayed on computer screen for 1000 ms. Participants were asked to typewrite the words after a recall signal (“Rappel”) displayed just after the last word disappeared. The WLE was observed, with short words being significantly better recalled (56%) than long words (51%) in correct position, *F*(1, 13) = 5.92, *p* < .05, *η^2^_p_*  = .31.

Bonin P, Méot A, Aubert LF, Malardier N, Niedenthal PM, Capelle-Toczek MC (2003) Normes de concrétude, de valeur d’imagerie, de fréquence subjective et de valence émotionnelle pour 866 mots. Annee Psychol 103: 655-694.
